# Deep Learning-Based Detection of Articulatory Features in Arabic and English Speech

**DOI:** 10.3390/s21041205

**Published:** 2021-02-09

**Authors:** Mohammed Algabri, Hassan Mathkour, Mansour M. Alsulaiman, Mohamed A. Bencherif

**Affiliations:** 1 Computer Science Department, College of Computer and Information Sciences, King Saud University, Riyadh 11543, Saudi Arabia; mathkour@ksu.edu.sa; 2 Center of Smart Robotics Research (CS2R), College of Computer and Information Sciences, King Saud University, Riyadh 11543, Saudi Arabia; msuliman@ksu.edu.sa (M.M.A.); mabencherif@ksu.edu.sa (M.A.B.); 3 Computer Engineering Department, College of Computer and Information Sciences, King Saud University, Riyadh 11543, Saudi Arabia

**Keywords:** articulatory features detection, object detection, YOLO, PER

## Abstract

This study proposes using object detection techniques to recognize sequences of articulatory features (AFs) from speech utterances by treating AFs of phonemes as multi-label objects in speech spectrogram. The proposed system, called AFD-Obj, recognizes sequence of multi-label AFs in speech signal and localizes them. AFD-Obj consists of two main stages: firstly, we formulate the problem of AFs detection as an object detection problem and prepare the data to fulfill requirement of object detectors by generating a spectral three-channel image from the speech signal and creating the corresponding annotation for each utterance. Secondly, we use annotated images to train the proposed system to detect sequences of AFs and their boundaries. We test the system by feeding spectrogram images to the system, which will recognize and localize multi-label AFs. We investigated using these AFs to detect the utterance phonemes. YOLOv3-tiny detector is selected because of its real-time property and its support for multi-label detection. We test our AFD-Obj system on Arabic and English languages using KAPD and TIMIT corpora, respectively. Additionally, we propose using YOLOv3-tiny as an Arabic phoneme detection system (i.e., PD-Obj) to recognize and localize a sequence of Arabic phonemes from whole speech utterances. The proposed AFD-Obj and PD-Obj systems achieve excellent results for Arabic corpus and comparable to the state-of-the-art method for English corpus. Moreover, we showed that using only one-scale detection is suitable for AFs detection or phoneme recognition.

## 1. Introduction

**Figure 1:**
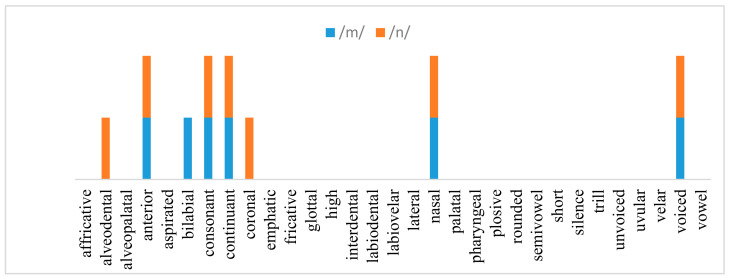
AFs of the two Arabic phonemes/m/and/n/. AF with no bar means this feature is absent in the two phonemes.

Speech consists of small units, called phonemes [1]. These phonemes are produced by the movement of the vocal tract parts (articulators), which are the tongue, lips, teeth, jaw, and velum [2]. Each phoneme has attributes or features describing their articulation. Based on these attributes, phonemes are described by binary vectors (e.g., ones and zeros) that indicate the existence and absence of articulatory features (AFs) [3,4]. For example, the phonemes/m/and/n/in the Arabic language have similar AF vectors, except at three AFs, where the alveodental feature exists in phoneme/n/and absent in phoneme/m/; bilabial feature exists in phoneme/m/and absent in a phoneme/n/; and/n/is a coronal, and/m/is not [3], as shown in Figure 1. AFs are used in studies related to pronunciation error detection [4,5], speech synthesis [6], speech pathology [7], tone recognition [8], and other speech domains. Reference [4] showed that AFs are robust against background noise and variations between speakers because of dialect, age, and gender [9]. Additionally, AFs are universal; hence, AFs of a specific language are common with many different languages [10]. In this context, several studies have been proposed to build universal (i.e., cross-language) speech recognition systems based on the universal property of AFs [11,12].

This paper presents an end-to-end system that detects the sequences of multi AFs from speech utterances by formulating the AF detection problem as an object detection problem. The proposed approach is inspired from our previous study [13], in which we proposed and demonstrated the effectiveness of using deep object detection techniques for phoneme recognition. Accordingly, this study investigates the application of a multi-label object detection technique for recognizing the sequences of multi-label AFs or phonemes of speech utterances. Our proposed systems successfully tackle the problem of AF and phoneme recognition and yield results better than those of the state-of-the-art techniques on the Arabic and English corpora. To the best of our knowledge, this is the first study that tackles the detection of an AF sequence using object detection techniques.

The rest of the paper is organized as follows: Section 2 summarizes the previous studies related to AF detection for the English and Arabic languages; Section 3 presents the theoretical background of methods of this study and the speech corpora; Section 4 presents the experimental results and discussion; and finally, Section 5 gives the conclusions and future work.

## 2. Literature Review

A review of the distinctive phonetic features (DPF) is presented in [14]. The DPF elements can be used to distinguish between phonemes, where each phoneme is represented by a vector of the presence and absence of these DPF elements. Based on data from Arabic linguistics and researchers, [14] showed the common DPF for modern standard Arabic. According to these DPFs, a recent study of modeling and extracting them using deep neural network and multi-layer perceptron is presented in [3]. Experiments were conducted using the KACST Arabic Phonetic Database (KAPD) corpus to extract the 31 DPF elements presented in a previous study [14]. A separate classifier for each DPF element was designed to extract the 31 binary values that represent the existence and absence of each DPF from 15 frames per phoneme. Each phoneme has a unique vector of DPFs; hence, the corresponding phonemes were extracted from the extracted vector of the DPFs, and the correct matching was calculated using the KAPD corpus test set. The first intensive study on the speech attribute detection for the Arabic language using DNN is presented in [15], in which the term speech attribute was used to correspond to the place and manner of articulation [4,15]. The authors designed a separate DNN classifier for each attribute from the 37 attributes considered. They used a corpus collected from the Holy Quran to evaluate the system. This corpus contained a 90 h recording. The data were automatically time labeled at the phoneme level. The reported results showed that the average accuracies were 84% and 83% for the place and manner of articulation, respectively.

Unlike the Arabic language, intensive studies on speech attribute detection have been made on the English language. We focused herein on studies related to the well-known corpus, TIMIT, because it is the corpus we used in our study. King and Taylor [16] proposed a speech recognition system based on phonological features instead of phonemes. They used a recurrent neural network to detect features from the continuous speech of TIMIT and performed three experiments. The first experiment was conducted to detect Chomsky–Halle binary features, called “sound pattern of English” [17], and reported an average of 92% correct frames for overall features. The second experiment focused on detecting six multi-valued features and trained a separate network for each of the six considered features. They obtained an average of 86% correct frames for overall features. The third experiment was performed for the Government Phonology (GP) [18]. Consequently, they achieved an average of 93% correct frames for overall features. More information about the GP and the TIMIT phoneme mapping to the GP are presented in [16]. Hou et al. [19] proposed an automatic speech attribute transcription system, which creates the probability attribute lattice based on a single frame or set of frames using an artificial neural network. They used 14 Chomsky–Halle speech attributes in their experiments and discussed the effect of unbalanced data caused by the difference between the number of present (+) frames and the number of absent (−) frames in the training set. The detection rates of these 14 speech attributes were as follow: less than 80% for eight of these attributes, greater than 90% for four of these attributes, and between 80 and 90% for the remaining two attributes. They proposed a balancing technique and reported detection rates greater than 90% for six attributes and greater than 80% for the remaining eight attributes.

Meanwhile, [4] proposed a pronunciation error detection system based on the anomaly detection problem to overcome the scarcity of the amount labeled as mispronounced speech using a one-class support vector machine (SVM). They used a binary DNN as a detector for each speech attribute (place and manner of articulation), then fed the output from all detectors to a one-class SVM to detect whether the phoneme was pronounced correctly or not. The system was trained using the TIMIT and WSJ0 corpora and tested using three corpora: a native corpus with artificial error, a foreign-accented corpus, and a corpus of children with disordered speech. 

From the above review, we found that most of methods used different classifiers for each AF. Each classifier takes the acoustic signal and produces a binary output that represents the existence or absence of the corresponding AF. This methodology is complex and has high computation power. To solve this issue, we investigated using object detection technique to have a system that detect all AFs, because the detector provides not only multi-label classification but also localization. This classification information is useful for detecting AFs or phonemes while localization is an additional information that is useful for other purposes such as segmenting the speech at the phoneme level and detecting the order of the phonemes.

## 3. Methods and Materials

This section presents the general idea of applying YOLOv3-tiny detector for AF and phoneme detection from the images of spectrogram of speech. Then, we give an overview of the proposed systems. Finally, the speech corpora used in this research are briefly described.

### 3.1. Adapting YOLOv3-Tiny Architecture for the Speech Application

The YOLO detector, which connotes the phrase “You Only Look Once,” is a one-stage object detector category developed by Redmon et al. [20]. The authors of the initial version, called YOLOv1, later proposed two modified versions for a total of three versions [20]. YOLOv3 has better performance than the preceding versions and supports multi-label detection; thus, we used it in our proposed system to detect the AF sequence of each phoneme in an utterance. We wanted our system to work in a real-time application; thus, we used the tiny version of YOLOv3 (YOLOv3-tiny). This section explains our adaptation of YOLOv3-tiny to detect the AF sequence. The original YOLOv3-tiny model consists of two-scale detection. Some authors improved this model by adding a third detection scale. Consequently, the results outperformed the original one [21,22,23,24]. YOLOv3-tiny consists of a small backbone network, called Darknet-Reference. The network was pre-trained using ImageNet and achieved 61.1% and 83.0% for the Top-1 and Top-5 accuracies, respectively. In terms of computation, it is faster than AlexNet, Resnet18, and VGG-16 [25] and consists of 13 convolutional and pooling layers. It only needs 0.96 billion floating-point operations per second, while AlexNet needs 2.27 billion operations [25]. Most object detection techniques in the literature use ImageNet pre-trained weights for the backbone network [26]. In our case, our images are not real images, but spectrogram images; hence, we investigated pre-training the backbone network for classifying the word of each spectrogram and used these weights to initialize the backbone weights of YOLOv3-tiny.

We will now explain how we used YOLOv3-tiny to detect the AFs within the spectrogram images. In the Arabic corpus, KAPD, the spectrogram width was 288 (number of frames), and its height was 32 (number of Mels). YOLOv3-tiny down-sampled the image to a factor of 32; thus, the grid cell of the first scale will be 9×1, and the number of predictions of each cell will be 108, where 108 = (31 (*# of classes*) + 5 (*box coordinates and confidence score*)] × 3 (*anchor boxes*)). 

Our spectrogram images were divided to nine consecutive cells. In the second scale, YOLO up-sampled the features measuring 9×1 to 18×2, then concatenated it with the feature of layer nine to make predictions with finer-grained information. Hence, the size of the grid cell of the second scale will be $\left(18\times 2\right)\;\mathrm{with}\;108$ predictions per cell. The same process is applied in the third scale, and the size of the grid cell of the third scale will be $\left(36\times 4\right)\;\mathrm{with}\;108$ predictions per cell. YOLOv3-tiny with two scales used six anchor boxes (three for each scale), while YOLOv3-tiny with three scales used nine anchor boxes (three for each scale). The anchor boxes were calculated using k-means clustering on the bounding boxes of the training data.

To this end, our objects (i.e., AFs or phonemes) had the same height; therefore, the center point of all objects falls in the middle of the spectrogram images. In each detection layer of YOLO, each grid cell was used to detect any object if the center of that object fits in this grid cell. Hence, we postulated that there is no need for the second and third scales to detect our objects because all our objects can be fitted to any of the grid cells of scale one. Accordingly, we proposed the YOLOv3-tiny model with only one-scale detection and only three prior anchors. We then compared its performance with those of the two and three scales. We faced the problem of the small number of possible detected objects by reducing the number of scales. To solve this issue, we increased the width of the input layer of the network from 288 to 576 for the Arabic KAPD corpus, which resulted in 18 grid cells of the detection layer instead of 9. For TIMIT, we increased the input layer width of the network from 2084 to 6144, resulting in 192 grid cells of the detection layer instead of 64. We called the three models from YOLOv3-tiny with one-, two-, and three-scale detection as YOLOv3-tiny-1S, YOLOv3-tiny-2S, and YOLOv3-tiny-3S, respectively. We then investigated their performance. Table 1 shows a comparison of the three used models in terms of the size of the input layer for each corpus, number of trainable parameters, and size of the trained model. The table shows that the model YOLOv3-tiny-1S had a lower number of parameters and a smaller size.

### 3.2. Proposed Systems

**Figure 2:**
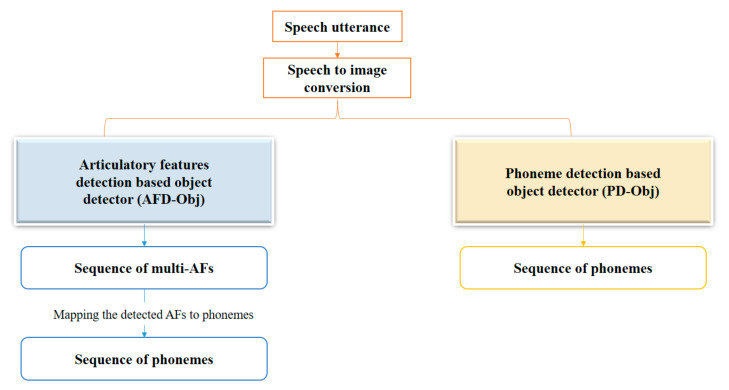
The proposed systems.

Our goal herein is to propose a light end-to-end system that would accurately detect the AF sequence within the whole utterance in real time. To achieve this goal, we propose the AFD-Obj system, which is a single network for AF sequence detection in Arabic and English speech. We do not use a different network for each AF, as done in some state-of-the-art systems [3,4,15], where a neural network is used for each AF to detect the presence or absence of an AF in speech frames or group of frames. We select the YOLOv3-tiny [27] detector because of its simplicity, fast computation property, and the fact that it supports multi-label detection. YOLOv3-tiny can process images at 220 frames per second (FPS) [28]. We also suggest a technique for converting the detector output to a form that can be used to calculate the detection accuracy of each AF. Each phoneme has a unique vector of AFs; hence, we can use our system for phoneme recognition by mapping the detected AFs to the corresponding phonemes, as suggested in Ref. [3]. Moreover, we propose PD-Obj, which is an end-to-end system for direct Arabic sequence phoneme recognition from the spectrogram without AF usage. Figure 2 shows the general overview of the proposed systems.

We study the effect of the number of scales in the detection layers of YOLOv3-tiny on AFD-Obj and PD-Obj by using three models: YOLOv3-tiny-1S, YOLOv3-tiny-2S, and YOLOv3-tiny-3S corresponding to one, two, and three scales, respectively. 

### 3.3. Speech Corpora

Three speech corpora were used herein: one was used to train the backbone network, and two were employed to train and test the proposed AFD-Obj and PD-Obj systems.

#### 3.3.1. Google Speech Commands Corpus (GC)

We used the Google speech command corpus to pre-train the Darknet-Reference network, which is the backbone network for the YOLOv3-tiny detector. Most of the CNN architectures in the literature have their weights trained using ImageNet, which is a large-scale image database consisting of millions of labeled images [29]. These weights were then used as pre-trained weights to investigate these architectures on small databases or different domains [30]. Many researchers have used ImageNet pre-trained weights in speech processing tasks that process spectrogram images [31,32]. The creators of the YOLOv3-tiny detector used Darknet-Reference as the backbone of their object detector and pre-trained its weights using ImageNet [33]. Moreover, the creators of SpeechYOLO [34] used the Google command database (V1 with classes = 30) to pre-train the CNN of their model. On the contrary, instead of using ImageNet, which is an image database, we investigated the initialization of the backbone network in a speech processing application and chose the Google speech command corpus (V2, with classes = 35) to pre-train the backbone network of the YOLOv3-tiny detector. The Google speech command corpus has two versions. We used the second version containing 105,829 audio files for 35 one-second words [35] to initialize the weights of Darknet-Reference by training the network to classify 35 words [35]. For validation and testing split, we used “*validaton_list.txt*” and “*testing_list.txt*” provided with the corpus [35].

#### 3.3.2. KACST Arabic Phonetic Database (KAPD)

We used the KAPD corpus to investigate the application of object detection techniques to detect Arabic AFs and recognize Arabic phonemes. KAPD was developed by King Abdul-Aziz City for Science and Technology at 2003 [36]. It contains the recording of seven native Saudi speakers for 1.2 h of recording. Yasser et al. [37] enhanced KAPD to fulfill the requirements of machine learning and data mining applications. They then used it to extract the Arabic DPF [3]. In our work, we used their version of KAPD, which was manually segmented into phonemes, and defined training and testing subsets. We randomly selected 10% from the training set for validation. Table 2 presents the Arabic phonemes, KAPD symbols, and IPA symbols with the number of occurrences of the training and testing samples of each phoneme used in our experiments.

**Figure 3:**
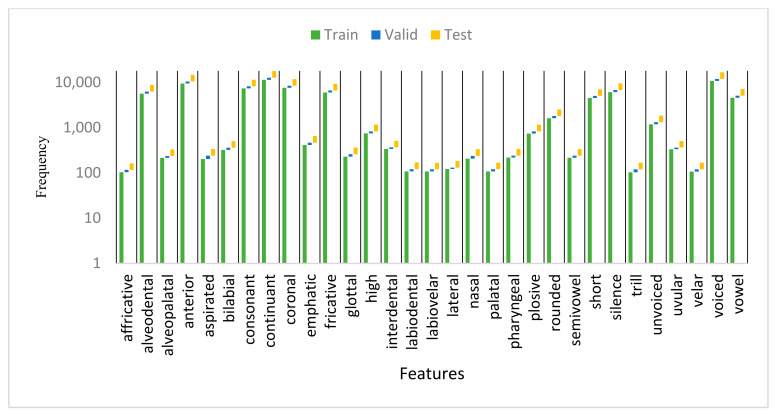
Occurrences of AF classes on the training, validation, and testing sets of KAPD.

They also provided the mapping table of the 34 Arabic phonemes plus silence to their corresponding 31 phonetic distinctive features [3]. We analyzed the number of occurrences of each of the 31 AFs in the training, validation, and testing sets of KAPD corpus in Figure 3.

#### 3.3.3. TIMIT Corpus

**Figure 4:**
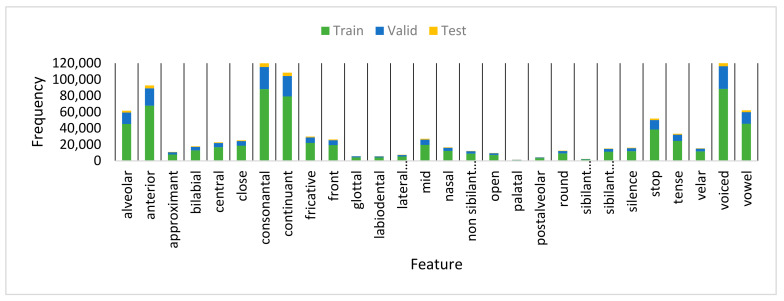
Occurrences of the AF classes on the training, validation, and testing sets of TIMIT.

We used TIMIT to evaluate our proposed system for AF detection in the English speech. TIMIT is a famous phonetic corpus extensively used in published work in phoneme recognition [38,39] and articulatory feature detection [40]. TIMIT consists of a recording of 640 speakers distributed between the training set (462 speakers), core test set (24 speakers), and complete test set (168 speakers) [41]. Ten sentences were uttered by each speaker. Similar to other studies in TIMIT, we did not consider the two dialect sentences (i.e., SA1 and SA2) in all experiments [42]. Studies on AFs using TIMIT differed in the number of AFs used. In this work, we selected one of the best state-of-the-art published work, presented in Interspeech 2019 [40] and followed the number of AFs and mapping between the TIMIT phonemes and the corresponding AFs. They used attention model to recognize the sequence of articulatory features for each utterance and 28 place and manner of articulations in their experiments. Figure 4 shows an analysis of the number of occurrences of each of the 28 AFs in the training, validation, and testing sets of the TIMIT corpus. We considered (h#, epi, and pau) phonemes as a silence attribute.

## 4. Experiments

### 4.1. Evaluation Metrics

We used different metrics to compare the performances of our proposed systems with those of the state-of-the-art baseline works in Arabic [3] and English [16,40] speech. Meanwhile, we used the metrics of [3] to calculate the AFD-Obj accuracy for the AF detection in Arabic speech. Imbalances were found between the number of existing and non-existing AFs in the training and testing sets, which yielded an imbalanced classification problem, as discussed in [3]. Hence, they used area under curve, geometric mean (GM), and F-measure as metrics for the imbalance situation. For simplicity, we used the GM and F-measure to evaluate the proposed system and deal with the imbalanced situation, as presented in Equations (2) and (3) [43]. We calculated the true positive (TP), true negative (TN), false positive (FP), and false negative (FN) for each AF element from the confusion matrix generated using the HResult module for each AF:

(1) $$GM=\sqrt{TPR\;.\;TNR}$$

(2) $$F_{measure\;}=\frac{2\left(TPR\;.\;PPV\right)\;}{PPV+TPR}$$

where:

$$TPR=\;\frac{TP}{TP\;+FN}\;FPR=\;\frac{FP}{FP+TN}$$

$$TNR=\;\frac{TN}{FP\;+TN}\;PPV=\;\frac{TP}{TP\;+FP}$$

We deduced the corresponding phonemes from the detected AFs and used the resulting phonemes to calculate the correction rate of the system through Equation (3):

(3) $$correction\;rate=\frac{N-S-D}{N}\times 100\%$$

where, N is the number of reference phonemes; S is the number of substitutions; and D is the number of deletions. For the PD-Obj system, we used the phoneme error rate (PER) calculated using Equation (4) [44]:

(4) $$PER=100\%-\left(\frac{N-S-D-I}{N}\right)\times 100\%$$

where, *N* is the number of reference phonemes; *I* is the number of insertions; *S* is the number of substitutions; and *D* is the number of deletions. We used the HResults tool to calculate the detection accuracy of the AFD-Obj system for the English speech, then compared it with that of the state-of-the-art methods [16,40].

### 4.2. Hardware and Software Specifications

The specifications of the machine used in conducting all experiments herein are: 64 GB (RAM), GeForce GTX 1080 Ti (GPU), and AMD Ryzen 16-Core Processor x 32 (CPU). We used the updated darknet repository for the deep learning framework [23] and the HResults module of HTK (version 3.4.1) for sequence alignments and PER.

### 4.3. Training and Testing of the Proposed Systems

**Figure 5:**
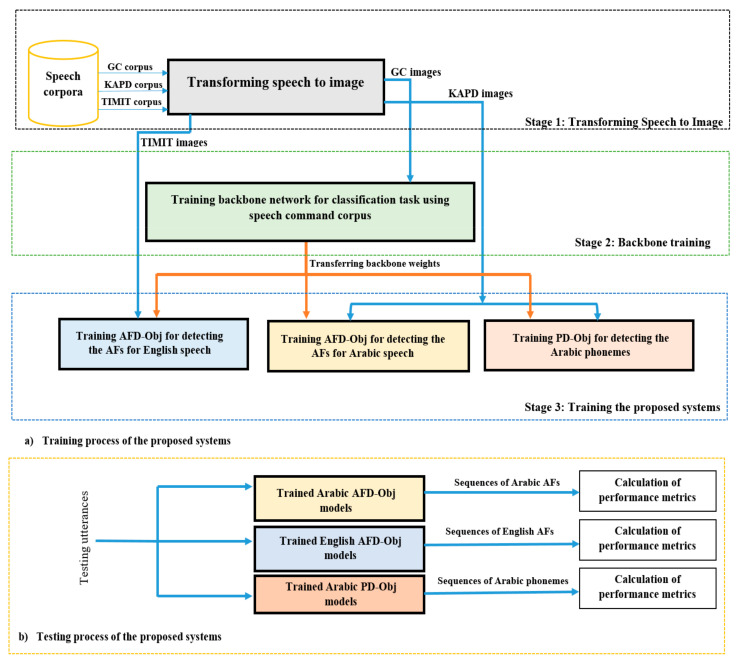
General overview of the proposed systems.

Figure 5 shows the training and testing processes of the proposed systems. The training phase consists of three stages (Figure 5a). In the testing phase (Figure 5b), the spectrogram images of the testing utterances are fed to the model of the corresponding system that recognizes and localizes the AF sequences or the phonemes. We give the details of each stage of the proposed systems in the following sections.

#### 4.3.1. *Stage 1*: Transforming Speech to Image

**Figure 6:**
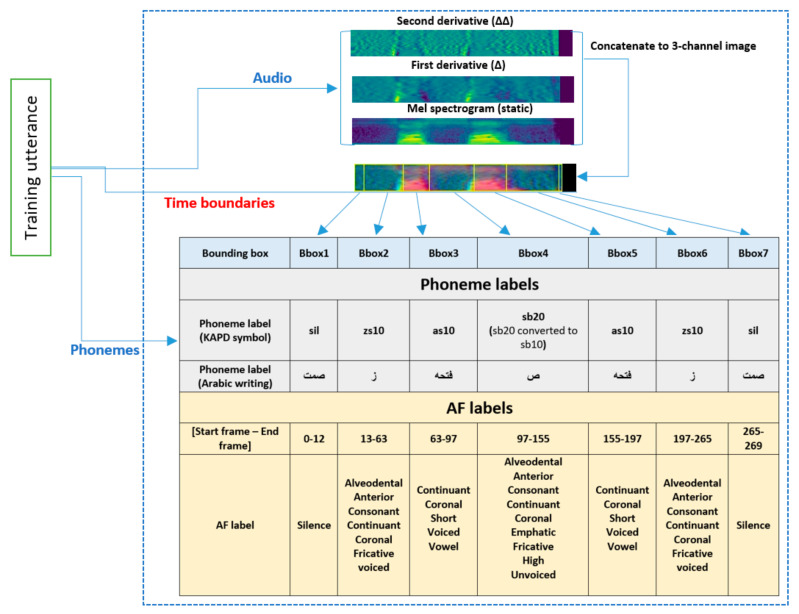
Example of creating a three-channel image and related bounding boxes.

The main motivation of this research was to formulate the AF detection problem as an object detection problem. Hence, we started by converting speech signals to spectrogram images and annotating images with the corresponding objects, where objects can be AF vectors or phonemes. We used the speech-to-image transformation presented in detail in our previous study [13]. Figure 6 shows an example of generating a spectral three-channel image from the speech and creating the associated bounding boxes for the utterance (GHSBGMA) from the KAPD training set. We concatenated the power Mel-spectrogram and the first and second derivatives to generate a three-channel image. Then, using the time boundaries, we calculate the bounding box of each object. The object in AFD-Obj has multi-labels where each label corresponds to a certain AF as shown in Figure 6. If the AF exists in the phoneme we added the label to this bounding box, otherwise we ignored that AF. For example, the following AFs (continuant, coronal, short, voiced, and vowel) exist in phoneme (as10) and the other 26 AFs do not exist, so, we annotated the third bounding box (Bbox3) by the following labels (continuant, coronal, short, voiced, and vowel). 

#### 4.3.2. *Stage 2*: Backbone Training

**Figure 7:**
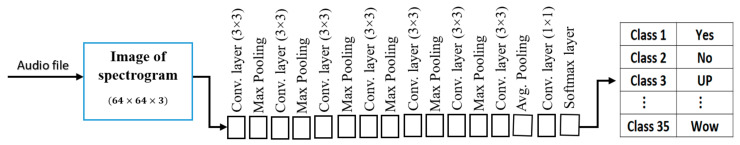
Darknet-Reference architecture.

The backbone network of the YOLOv3-tiny detector is the Darknet-Reference presented in Ref. [33]. The network consists of 13 consecutive convolutional and max-pooling layers. Ref. [45] showed that the swish activation function proposed by Google Brain outperformed LReLU used in the original backbone network and other activation functions; hence, we used the swish activation function. We trained this network using the Google speech command corpus (V2) for the command classification task using two model names: Darknet-Reference-Leaky and Darknet-Reference-Swish. We used 35 commands to train and evaluate the network (Figure 7). The length of each audio clip was 1 s. All utterances were sampled at 16 KHz. The total number of samples for training, validation, and testing was 84,843, 9981, and 11,005, respectively. We generated a log Mel-spectrogram using 25 ms frame length, 16 ms frame stride, and 64 Mels to convert speech to image. The delta and delta–delta were then computed and concatenated to create three-channel images. The output of this phase was a square image with a (64 × 64 ×3) dimension. We used the following training parameters: 0.01 learning rate, 128 batch size, 0.9 momentum, 0.0005 decay, and 50,000 number of iterations. The learning rate was reduced by 10× when the number of iterations reached 30,000 and 40,000. We used 96 and 32 as the maximum and minimum cropping sizes for cropping data augmentation, respectively.

We achieved the validation accuracy of 94.7% on the validation set. The network weights without the fully connected layers were used as the pre-trained weights in our system training. Our goal from this step was only to train the backbone network using spectrogram images instead of ImageNet images rather using trained weights to initialize the backbone layers of the following steps. Fortunately, we achieved results, in this specific task, comparable with state-of-the-art models considering the simplicity of our network. Table 3 summarizes the accuracy of our model compared with that of the state-of-the-art techniques [46,47,48].

The result of our models was better or close to the published state-of-the-art results in Refs. [46,47,48] for the same corpus (V2) and task (35-commands). 

#### 4.3.3. *Stage 3*: Training the AFD-Obj and PD-Obj Systems

In this stage, we trained the three YOLOv3-tiny models of the proposed AFD-Obj system to detect the AF sequence from spectrogram images. Similarly, we trained the three YOLOv3-tiny models of the proposed PD-Obj system to detect the phoneme sequence from spectrogram images.

(1) Training AFD-Obj for the Arabic corpus

This section presents the details of the training process for detecting the multi-label AFs in the continuous Arabic speech. We set the yolo layer configuration as follows: classes = 31 (i.e., number of articulatory features in the KAPD); random = 0 (because all images had the same size); the number of filters in each convolutional layers before the yolo layer is 108 ($31\;\left(class\right)+5\;\left(predicted\;elements\right)\times 3\;(number\;of\;anchors$), and jitter = 0.1. We disabled all augmentation parameters except jitter. For the YOLOv3-tiny-3S and YOLOv3-tiny-2S models, we set the network width to 288 and 448, respectively. That for the YOLOv3-tiny-1S model was set to 576.

(2) Training AFD-Obj for the English corpus

This section presents the details of the training process to detect the AF sequence using the TIMIT English corpus. We followed the same training procedure in the previous section, except for the YOLO layer configuration and the network dimension to conform to the number of TIMIT AFs. We used these parameters for the YOLO layers: classes = 28 (i.e., number of AFs in the TIMIT corpus); random = 0 (because all images had the same size); and the number of filters in each convolutional layers before the yolo layer is 99 ($28\;\left(class\right)+5\;\left(predicted\;elements\right)\times 3\;(number\;of\;anchor$). In terms of the network width and height, we used (6144, 32), (4096, 32), and (2048, 32) for the YOLOv3-tiny-1S, YOLOv3-tiny-2S, and YOLOv3-tiny-3S models, respectively.

(3) Training PD-Obj for the phoneme recognition in the Arabic corpus

We present the details of the training process of the proposed models for the Arabic phoneme detection using the PD-Obj system. This system has some similarities to that used in our previous study, which was recently published in [13]. The differences between this system and the previous one can be summarized in the following points: first, we did not use ImageNet weights as the pre-trained weights for the backbone network in the previous system [13]; in this system, we trained the backbone network from scratch using spectrogram images; second, in this system, the investigation used the one- and two-scale YOLOv3-tiny detector plus the three-scale one used in our previous work. Finally, we applied the proposed system in an Arabic corpus different from that used in our previous study to compare the performance of our system with that of the system used in [3], which was studied using AFs for phoneme recognition. The KAPD corpus was used to train and test the proposed models. The total number of phonemes was 35 (34 Arabic phonemes + silence). 

#### 4.3.4. Testing AFD-Obj System

The AFD-Obj system input was a three-channel image representing the whole utterance, while the output comprised the detected AF vectors and the location of each AF vector. One or more AFs may exist for each frame. Our proposed system detected the AFs for each frame based on the coordinates of the detected bounding boxes on the three-channel image of the spectrogram.

For the Arabic speech, we calculated the system performance at the phoneme level (i.e., existence of each AF for each phoneme) to compare with the system for the Arabic speech in [3]. We dealt with each detected bounding box as one detection to calculate the accuracy at the phoneme level and calculated the accuracy of the detected output compared with the canonical output. The output of our proposed AFD-Obj system was a sequence of detected bounding boxes. Each box contained the existing AFs. The lengths of these sequences had different sizes compared to the canonical. Therefore, we cannot directly calculate the accuracy of the detected output. To tackle this issue, we propose the use of the sequence alignment algorithm between the detected and canonical output. We did this alignment using the HResult analysis tool of HTK. HResult uses dynamic programming to make the sequence alignment [44]. We can calculate the number of correct detection and the number of insertion, deletion, and substitution errors by using the HResult output.

**Figure 8:**
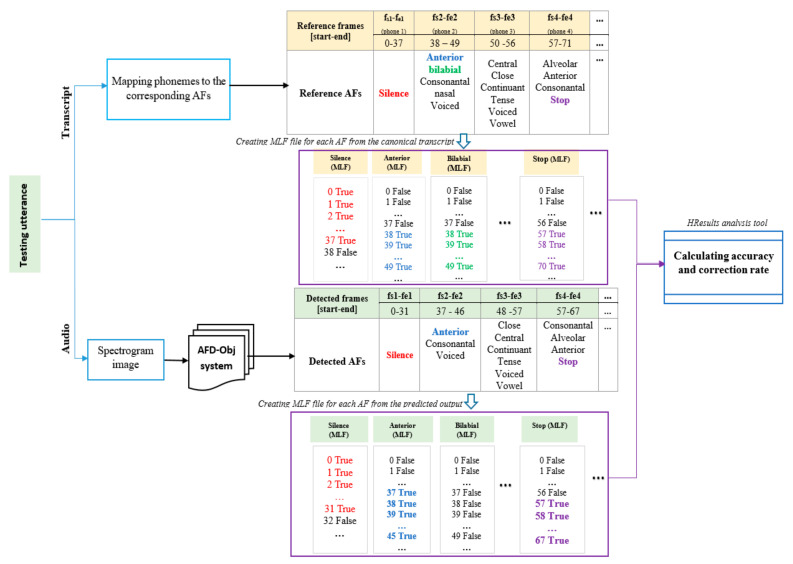
Testing phase of the AFD-Obj system: calculate the frame level accuracy of the detected outputs.

For the English speech, we calculated the performance of our proposed system at the frame level, as in [16,40]. We derived the canonical AFs and their timing from the phoneme transcript and the associated timing (available for TIMIT) (first step, Figure 8) to calculate the accuracy of detecting AFs at the frame level. The process of deriving the AFs from the phonemes then followed [16].

We created a multiple-label file (MLF), which was required by the HResult tool, for each AF from the canonical and detected output (i.e., output of AFD-Obj). We marked the frames of this period as True in the MLF file when an AF exists from the start frame (f_s_) to the end frame (f_e_), otherwise, frames were marked as False. We then performed the alignment and calculated the performance. Therefore, using the alignment procedure, we made sure that all predicted and canonical frames were taken in the performance analysis. Figure 8 shows the general overview of the testing phase of the AFD-Obj system.

**Figure 9:**
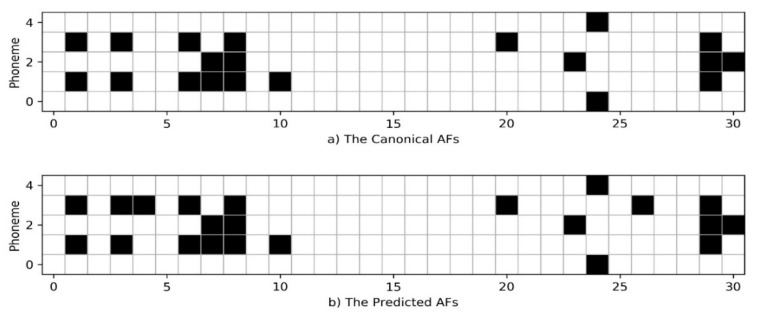
Visualization of the AFs in the test file “CYDSSFA” from KAPD corpus. (**a**) The canonical AFs. (**b**) the predicted AFs.

Figure 9 shows the visualization of the reference AFs and the output of our proposed AFD-Obj system using YOLOv3-tiny-1S model, for the CYDSSFA file from the test set of KAPD corpus. The utterance consists of five phonemes which are (/sil/,/zs10/,/as10/,/ds10/, and/sil/). We can clearly see that our system can predict all AFs of all phonemes correctly except the AF features number 4 and 26, which are AF aspirated and AF unvoiced, respectively. 

#### 4.3.5. Testing PD-Obj System

We followed the same procedure in our previous work to test the PD-Obj system [13]. We processed the detector output by removing the duplicate detection using the non-maximum suppressing algorithm. We then calculated the PER between the sequences of the detected phoneme and the canonical one.

### 4.4. Results and Discussion

This section presents and discusses three points: Firstly, we present the results of the proposed AFD-Obj system for detecting AF sequences in Arabic and English speech using three variations of the YOLOv3-tiny detector. Secondly, we show the performance of the proposed PD-Obj system for detecting the Arabic phoneme sequence. Thirdly, both system performances are compared to state-of-the-art methods.

#### 4.4.1. Results of AFD-Obj for Detecting AFs in Arabic Corpus

Table 4 shows the GM and F-measure for the Arabic AF detection using the proposed AFD-Obj system with the three proposed YOLO models (i.e., YOLOv3-tiny-1S, YOLOv3-tiny-2S, and YOLOv3-tiny-3S). For all AFs, the systems achieved a GM greater than 80%, except for labiodental. For the F-measure of all AFs, the systems achieved accuracies greater than 80%, except for labiodental, which had an F-measure of 72.1%, 77.6%, and 72.9% using YOLOv3-tiny-1S, YOLOv3-tiny-2S, and YOLOv3-tiny-3S, respectively, and interdental, which had an F-measure of 77.5% using YOLOv3-tiny-1S. In general, we achieved GM and F-measure average accuracies of 96.5% and 94.1% for the YOLOv3-tiny-1S model, 96.4% and 94.3% for the YOLOv3-tiny-2S model, and 96.4% and 94.5% for the YOLOv3-tiny-3S model. These results are better than those of state-of-the-art results [3], where approximately 45% of the AFs obtained less than 80% for GM and approximately 61% obtained less than 80% for the F-measure using their best model (i.e., DBN–DNN). We achieved our results using a single network for all AFs, while Ref. [3] used a different network for each AF. Moreover, our testing input is a whole utterance without time boundary information, while theirs was speech phonemes. We also detected the time boundaries of each AF; therefore, we can calculate the accuracy at the frame level.

- Extraction of the Arabic phonemes from the detected AFs

**Figure 10:**
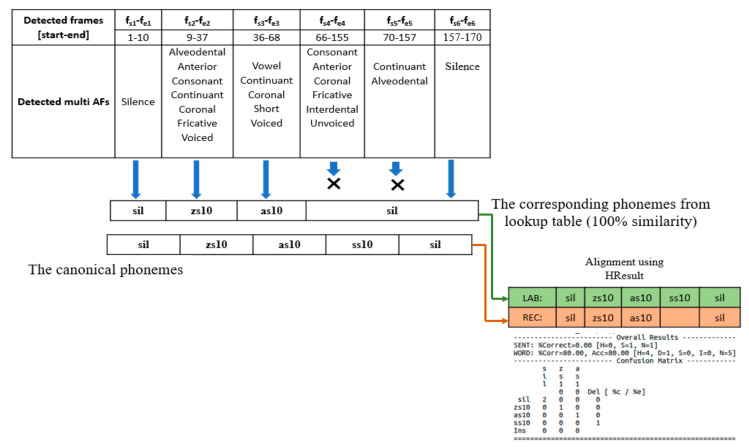
Testing example of converting the detected AFs using the YOLOv3-tiny-1S model to the corresponding phonemes and calculating the percentage of correct phonemes using the HResults tool (file “CMSSSFA”) from the KAPD corpus test set. X sign means invalid output, which occurs when the minimum hamming distance is greater than threshold (threshold = zero in case of 100% similarity).

Each phoneme has a unique vector representing the existences or absences of each AF; thus, we can detect the phonemes and their boundary from the AF vectors of each frame. We used the lookup table provided in Ref. [3] to produce the corresponding phoneme from the vectors of the detected AFs. Ref. [3] used the correct matching rate metrics for their evaluation. However, the output of our proposed system is a sequence of AFs; hence, the length of the output sequence is not uniform with the canonical form, and we calculated the correction rate and the PER by applying the sequence alignment between the matching output of the detected vectors and the canonical phonemes. Figure 10 shows an example of recognizing the phonemes from the detected AF vectors and calculating the correction rate.

We considered only the phonemes when there is an exact match (i.e., ideal case) of 100% similarity between the predicted AF vector and the reference vector, which might yield substitution errors, using zero hamming distance and ignored the invalid output. Ref. [3] reported the result for a 3-bit difference between the detected AFs and the lookup table, which amounted to approximately 90% similarity between the predicted and actual vectors. We compared the correction rate of our proposed method and that of [3] using the 100% and 90% similarities in Table 5. For our best model (i.e., YOLOv3-tiny-1S), we outperformed the matching rate of their best classifier (i.e., DBN–DNN) by almost 40% at 100% similarity and outperformed the matching rate of their classifier by approximately 4% at 90% similarity [3]. Using 100% similarity, we achieved correction rates of 86.04%, 88.06%, and 89.35% for YOLOv3-tiny-3S, YOLOv3-tiny-2S, and YOLOv3-tiny-1S, respectively, compared to 64% (matching rate) for the model in Ref. [3]. These values increased to 91.16%, 92.38%, and 92.59%, respectively, when using 90% similarity for all three models compared to 89% for that in [3]. This increase can be attributed to the fact that the correction rate measure ignored the insertion errors; hence, we ignored many insertion errors when using only 90% similarity.

For 100% similarity, our models obtained PERs of 14.13%, 12.09%, and 10.84%, respectively, which increased to 20.1%, 15.53%, and 12.57%, respectively, for 90% similarity (Table 5). Reference [3] did not provide the PER result. Another point to highlight is that these observations confirmed our postulation for not needing the second and third scales of the YOLO detector in the AF detection and phoneme recognition. The PER results also illustrate that using 90% similarity during AF matching to generate the corresponding phonemes is not acceptable because more wrong phonemes can be recognized as correct.

**Figure 11:**
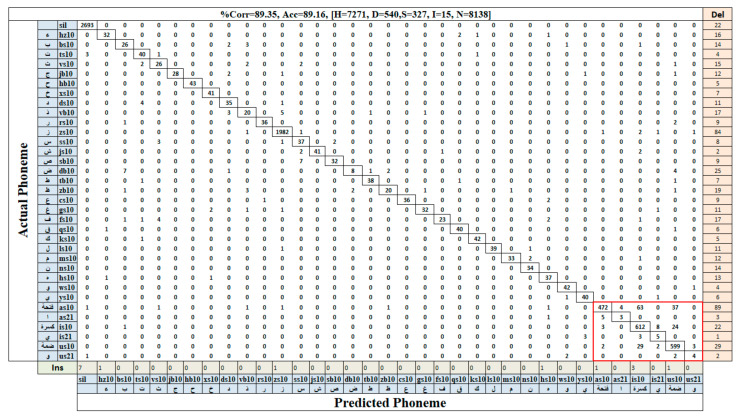
Confusion matrix of the detected Arabic phonemes from matching AF vectors using the AFD-Obj (YOLOv3-tiny-1S) model at 100% similarity.

Figure 11 shows the confusion matrix of the Arabic phonemes of the KAPD corpus generated from the detected AF vectors using our best model, YOLOv3-tiny-1S, with 100% similarity. We can clearly see from the confusion matrix that most phonemes were recognized correctly, and most of the confusion occurred between the phonemes with almost similar AF vectors. For example, phoneme /fs10/ was wrongly recognized for 4 times as /vs10/. Both phonemes had a similar AF vector, differing only in three elements:/vs10/ is coronal, interdental, and not labiodental, while /fs10/ is labiodental, not coronal, and not interdental. Our system also wrongly recognized phoneme /as10/ as /is10/ for 63 times. The AF vector for these phonemes showed that the two phonemes differed only in two AFs: phoneme /as10/ is coronal, and /is10/ is not; /is10/ is anterior, and /as10/ is not.

Another interesting observation is that the number of confusion is high for the short vowels (i.e.,/as10/,/is10/,/us10/) as highlighted by the red box in the Figure 11. Most of the confusion occur between these three phonemes. For example, /as10/ is wrongly recognized 63 times as /is10/ and 37 times as /us10/. This is expected since vowels are near to each other than other phonemes. The high number of confusions for these short vowels is due to their high number in testing data compared to other phonemes, except (/sil/ and /zs10/). Another observation is that the confusion of those vowel phoneme is very small with the non-vowel phonemes. For examples, /as10/ phoneme is wrongly recognized 6 times as non-vowel phonemes which represent around (1%) of all /as10/ detection. 

In addition, we can see that the number of the deletion errors is high compared to insertion and substitution errors. We attribute this to our requirement of 100% similarity matching which resulted in ignoring a lot of the detected AFs resulting in high number of deletions.

#### 4.4.2. Results of the AFD-Obj System for Detecting AFs in the English Corpus

This section presents the results of applying our proposed system for detecting the English AFs using the TIMIT corpus. We used accuracy at the frame level, by considering the bounding box coordinates as the start and end frames, as our evaluation metric, which is calculated as shown in Figure 8. We then compared the results of the proposed system with that of the state-of-art published work in AFs detection using TIMIT [40], called LAS-MTL-M. We consider for our comparison the results of LAS-MTL-M which were reported at frame level. Authors of [40] used the TIMIT segments markup (time boundaries) to calculate the accuracies of the column “markup frames” and the DTW algorithm to convert soft attention to hard attention to calculate the accuracies of the “frames” column in Table 6. In both cases, they dealt with the different number of predicted and target frames by taking the minimum length of target and prediction, as shown in the code provided. For better comparison, we calculated the accuracy of our proposed system using the coordinates of the detected bounding boxes as markup frames, after taking the minimum length of the predicted and target frames. This result is presented in the column “bounding box coord.” of Table 6. We also used another measure to deal with the problem of the difference in length between the predicted and target frames, where we used HResults analysis tool to align the predicted and target frames, then we calculate the accuracy. Moreover, HResults accuracy is more precise because it considers the insertion errors. This accuracy is presented in column “HResult align.” of Table 6**.** We also show the results with those in [16], which worked on detecting only some AFs in TIMIT.

Table 6 presents the result of our proposed system AFD-Obj with the three models. The table shows that our system achieved results comparable to the published result for all TIMIT AFs. Our models had an average accuracy (with bounding box coord.) of 94.29%, 95.04%, and 95.13%, and average accuracy (using HResult) of 93.23%, 93.47%, and 93.66% for YOLOv3-tiny-3S, YOLOv3-tiny-2S, and YOLOv3-tiny-1S, respectively, while the phones-las-frames model had an average detection accuracy of 95.5% using markup-frames. Since the test results of the “markup-frames” of [40] depend on segmenting the speech into markup frames, while our system doesn’t depend on any segmentation, hence we think fair comparison should be with the result of the “frames” column of [40].

An important observation from Table 6 is that our models detected silence within the utterance with a high accuracy compared to the phone-las models [40], which achieved only 63% and 80% for frames and markup frames, respectively. This high performance in detecting silence in continuous speech is very promising and can be looked at as an important achievement by itself. YOLOv3-tiny-1S had the best average detection accuracy (95.13%), while our model YOLOv3-tiny-2S had almost the same average detection accuracy (95.04%). The average detection accuracy of YOLOv3-tiny-3S was 94.29%. These results reinforce our previous assumption for not needing the second and third scales of YOLO detection for our specific application.

#### 4.4.3. Results of PD-Obj for the Phoneme Recognition in the Arabic Corpus

Table 7 presents the results of investigating the proposed PD-Obj system for phoneme recognition using the KAPD corpus. Our proposed system using the YOLOv3-tiny-2S model achieved the lowest PER of 5.63%, while the YOLOv3-tiny-1S and YOLOv3-tiny-3S models achieved 5.79% and 6.29% PER, respectively. These results are remarkable and show that our proposed system has an excellent potential compared to the recent state-of-the-art systems on this corpus [49] (last row, Table 7). Reference [49] used the HMM for the Arabic phoneme recognition using the DPF elements. The results also reinforced our previous assumption for not needing the second and third scales of the YOLO detection for our specific application.

We observe from Table 7 that the PD-Obj system obtained better results than the system based on the AFD-Obj. However, detecting the AFs is important in many applications such as, pronunciation error correction and diagnosis. And the AFs are universal between many languages. An interesting point for future work is to see how to improve the accuracy of the system that performs phoneme recognition based on the detected AFs.

**Figure 12:**
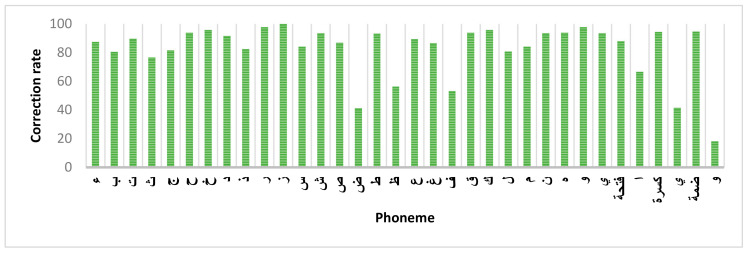
Correction rate of an Arabic phonemes using YOLOv3-tiny-1S model.

We also calculated the correction rate of each phoneme for the YOLOv3-tiny-1S model (Figure 12). Note that 79% of the Arabic phonemes had a correction rate greater than 80%, while 44% had a correction rate greater than 90%.

**Figure 13:**
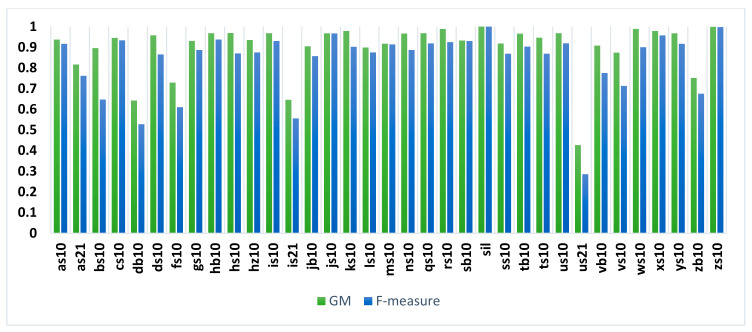
Geometric mean (GM), and F-measure of an Arabic phonemes of the KAPD corpus using YOLOv3-tiny-1S model.

KAPD corpus is imbalanced as shown in Table 2. In particular, the samples for /sil/, which is not a phoneme, and phoneme /zs1/ represent around half of the training and testing samples. To deal with imbalance the GM and F-measure for each phoneme are calculated from confusion matrix and are shown in Figure 13, after excluding the insertions and deletions. When we analyzed the performance of the proposed method in Figure 13 with the total number of samples of each phoneme, we observed that there is no direct relationship between the number of samples and the GM and F-measure. For example, we achieved a GM of 99% for /rs10/ and /ws10/ and 94% for /as10/ while the total number of samples is 168, 167, and 2352 for the phonemes /rs10/, /ws10/, and /as10/, respectively.

Table 8 shows the performance for the speech of each speakers from the test set of the KAPD corpus. The test set consists of the speech of the two speakers (M and Y) from each group (C and G), as presented in [37]. The total number of utterances and phonemes of the test sets are 1360 and 8138, respectively. We can notice from the results of Table 8 that our proposed system achieved excellent results. The best PER (lower) was obtained for speaker/subset Y/C, which is 4.4 with correction rate of 95.8%. The average and standard deviation of PER of all speakers are 5.8 and 1.2, respectively. 

Moreover, we reported in our previous study [13] that our proposal of using object detection techniques for phoneme recognition achieved excellent results in phoneme recognition using TIMIT. These results were comparable to all and better than those of some state-of-the-art techniques.

## 5. Conclusions

In this study, we successfully applied a state-of-the-art multi-label deep object detection technique for detecting AF sequences in the Arabic and English languages from the speech spectrogram. We selected the YOLOv3-tiny detector because of its real-time property and its support of multi-label detection. We investigated our proposed AFD-Obj system with different YOLOv3-tiny models. The first model YOLOv3-tiny-2S is the original version of the YOLOv3-tiny detector that performed a two-scale detection. The optimized version of the YOLOv3-tiny detector with three-scale detection was investigated. Finally, we proposed the usage of only one-scale detection in our proposed model (i.e., YOLOv3-tiny-1S). We have experimentally shown that using YOLOv3-tiny-1S would be suitable for AF detection or phoneme recognition. 

We used two corpora to examine the performance of the proposed models for AF detection in Arabic and English speech. We employed the KAPD corpus, which provides segmented data at the phoneme level, to detect the AFs in Arabic speech and the TIMIT corpus, which also has segmented data at the phoneme level, for the English AF detection. We then compared the performance of our method with those of the state-of-the-art methods using the two corpora of the two languages and consequently obtained better results. We also showed that our system, AFD-Obj, can detect the AFs at the frame level by decoding the output of the object detector. Moreover, we successfully applied the proposed PD-Obj system for the Arabic phoneme recognition and achieved a PER of 5.63% and 5.79% using the YOLOv3-tiny-2S and YOLOv3-tiny-1S models, respectively.

Our proposed models obtained remarkable results for KAPD, for AFs and phoneme detections, compared to the state-of-the-art methods. For AFs detection in TIMIT, our results were comparable to state-of-the-art published results. Moreover, they are light and suitable for real-time speech processing application. 

An advantage of our systems is that they are end-to-end, where the input is a whole utterance, and the output is a sequence of detected AFs or phonemes.

In this study, we used the detector output as is; hence, post-processing can be applied in the future work and might improve the results. Accordingly, anchor-free detectors can be investigated as a future work.

## Figures and Tables

**Table 1:** Characteristics of the YOLOv3-tiny models.

Corpus	Model	Network Resolution	# of Parameters (M)	Size of Trained Model (MB)
KAPD	YOLOv3-tiny-3S	288×32	9.1	36.4
	YOLOv3-tiny-2S	448×32	8.75	35
	YOLOv3-tiny-1S	576×32	7.8	31.2

**Table 2:** KAPD phonemes and distribution.

Arabic Phoneme	KAPD Symbol	Training Samples	Testing Samples	Arabic Phoneme	KAPD Symbol	Training Samples	Testing Samples
sil	sil	6767	2715	ع	cs10	121	48
ء	hz10	128	52	غ	gs10	123	48
ب	bs10	121	47	ف	fs10	121	49
ت	ts10	120	49	ق	qs10	120	48
ث	vs10	122	48	ك	ks10	120	48
ج	jb10	117	45	ل	ls10	131	52
ح	hb10	120	48	م	ms10	119	48
خ	xs10	120	48	ن	ns10	119	48
د	ds10	123	51	ه	hs10	131	52
ذ	vb10	121	47	و	ws10	120	47
ر	rs10	120	48	ي	ys10	121	48
ز	zs10	5169	2073	فتحة	as10	1681	671
س	ss10	121	51	ا	as21	30	12
ش	js10	120	48	كسرة	is10	1666	670
ص	sb10	117	48	ي	is21	19	12
ض	db10	106	48	ضمة	us10	1667	664
ط	tb10	119	48	و	us21	20	11
ظ	zb10	123	48	Total		20,283	8138

**Table 3:** Accuracy of the test set of the Google command database (V2) using 35 cmds.

Model	Testing Accuracy
Attention_CRNN [46]	93.9%
SampleCNN [47]	94.82%
TR-CRNN [48]	96.00%
Darknet-Reference-Leaky (our)	94.32%
Darknet-Reference-Swish (our)	94.49%

**Table 4:** Performance metrics of the proposed system AFD-Obj using three models (i.e., YOLOv3-tiny-1S, YOLOv3-tiny-2S, and YOLOv3-tiny-3S) for the Arabic AFs.

	YOLOv3-Tiny-1S		YOLOv3-Tiny-2S		YOLOv3-Tiny-3S	
	GM	F-Measure	GM	F-Measure	GM	F-Measure
affricative	0.929	0.927	0.931	0.929	0.931	0.929
alveodental	0.988	0.982	0.989	0.986	0.992	0.989
alveopalatal	0.938	0.936	0.927	0.925	0.945	0.943
anterior	0.980	0.982	0.985	0.986	0.989	0.990
aspirated	0.988	0.907	0.978	0.918	0.994	0.941
bilabial	0.954	0.876	0.930	0.868	0.940	0.908
consonant	0.998	0.998	0.997	0.997	0.999	0.998
continuant	0.992	0.993	0.994	0.994	0.993	0.994
coronal	0.977	0.975	0.980	0.978	0.984	0.983
emphatic	0.904	0.891	0.912	0.900	0.913	0.904
fricative	0.992	0.990	0.993	0.991	0.990	0.990
glottal	0.968	0.903	0.984	0.915	0.975	0.933
high	0.932	0.918	0.939	0.923	0.927	0.920
interdental	0.856	0.775	0.865	0.811	0.879	0.833
labiodental	0.795	0.721	0.838	0.776	0.803	0.729
labiovelar	1.000	0.967	0.988	0.953	0.988	0.966
lateral	0.960	0.922	0.960	0.897	0.969	0.873
nasal	0.979	0.963	0.951	0.929	0.978	0.973
palatal	0.978	0.967	0.978	0.977	0.967	0.945
pharyngeal	0.984	0.984	0.966	0.960	0.967	0.961
plosive	0.960	0.913	0.961	0.926	0.965	0.936
rounded	0.982	0.940	0.987	0.949	0.990	0.966
semivowel	0.989	0.967	0.994	0.983	0.989	0.972
short	0.997	0.995	0.999	0.998	0.999	0.998
silence	0.999	0.999	0.998	0.998	1.000	0.999
trill	0.955	0.933	0.954	0.932	0.919	0.874
unvoiced	0.985	0.964	0.983	0.972	0.982	0.971
uvular	0.960	0.917	0.953	0.932	0.938	0.926
velar	0.989	0.958	0.968	0.928	0.989	0.948
voiced	0.995	0.996	0.996	0.996	0.996	0.997
vowel	0.999	0.999	1.000	0.999	0.999	0.999
Average	0.965	0.941	0.964	0.943	0.964	0.945

**Table 5:** PER (%) and correction rate (%) for our proposed AFD-Obj system and results of [3].

Matching Rate (# Bits)	Model	PER (%)	Correction Rate (%)
100% (0 bit)	YOLOv3-tiny-3S	14.13	86.04
	YOLOv3-tiny-2S	12.09	88.06
	YOLOv3-tiny-1S	10.84	89.35
	DBN-DNN [3]	-	64.00 (Exact matching rate)
90% (3 bits)	YOLOv3-tiny-3S	20.1	91.16
	YOLOv3-tiny-2S	15.53	92.38
	YOLOv3-tiny-1S	12.57	92.59
	DBN-DNN [3]	-	89.00 (Matching rate)

**Table 6:** Detection accuracy of all 28 English AFs using the proposed system AFD-Obj and state-of-the-art methods.

Articulatory Features	AFD-Obj System						LAS-MTL-M Markup-Frames [40]	LAS-MTL-M Frames [40]	KT [16]
	YOLOv3-Tiny-1S		YOLOv3-Tiny-2S		YOLOv3-Tiny-3S				
	Bounding Box Coord.	HResult Align.	Bounding Box Coord.	HResult Align.	Bounding Box Coord.	HResult Align.			
Alveolar	91.05	90.22	90.92	90.01	89.31	88.96	95	77	
Anterior	89.69	89.34	89.55	89.02	87.92	88.08	90	69	90
Approximant	97.12	95.39	97.17	95.32	96.87	95.39	98	94	68
Bilabial	97.70	95.89	97.53	95.57	97.30	95.78	98	93	
Central	93.73	92.31	93.73	92.18	93.36	92.27	99	91	
Close	94.13	92.65	94.02	92.46	93.36	92.33	97	88	86
Consonantal	88.97	88.75	88.75	88.42	87.32	87.64	88	64	90
Continuant	91.37	90.46	90.88	90.04	88.60	88.38	89	68	86
Fricative	96.03	94.56	95.73	94.21	95.04	94.06	95	83	88
Front	93.33	91.96	93.42	91.92	92.06	91.12	95	89	84
Glottal	98.67	96.69	98.62	96.48	98.42	96.82	99	98	
labiodental	98.88	96.89	98.80	96.71	98.57	96.94	99	96	
Lateral approximant	98.21	96.34	98.11	96.07	97.88	96.31	99	96	
Mid	90.28	89.09	90.04	88.77	88.87	88.3	97	82	
Nasal	97.59	95.95	97.55	95.72	97.15	95.74	99	93	84
Non sibilant fricative	97.60	95.8	97.50	95.58	97.22	95.74	97	94	
Open	96.09	94.31	95.83	93.98	95.63	94.23	98	91	93
palatal	99.60	97.54	99.63	97.4	99.57	97.81	99	99	
postalveolar	99.18	97.12	99.17	96.94	98.96	97.21	99	97	
Round	94.99	93.36	94.70	92.97	94.30	93.04	98	91	92
Sibilant affricate	99.50	97.41	99.51	97.29	99.38	97.64	99	99	
Sibilant fricative	97.97	96.1	97.81	95.95	97.37	96	98	90	
Silence	96.79	95.21	97.05	95.29	96.68	95.35	80	63	89
Stop	95.03	93.74	95.05	93.61	94.46	93.53	97	85	96
Tense	89.63	88.46	89.92	88.73	88.65	87.94	97	81	87
Velar	98.37	96.47	98.31	96.25	98.01	96.37	99	95	
Voiced	90.86	89.9	90.71	89.69	88.62	88.19	84	72	93
vowel	91.31	90.69	91.19	90.57	89.29	89.26	92	70	92
Average	95.13	93.66	95.04	93.47	94.29	93.23	95.5	86	

**Table 7:** PER and correction rate of the Arabic phoneme recognition using the proposed models.

Model	PER (%)	Correction Rate (%)
PD-Obj (YOLOv3-tiny-3S)	6.29	93.94
PD-Obj (YOLOv3-tiny-2S)	5.63	94.56
PD-Obj (YOLOv3-tiny-1S)	5.79	94.34
AFD-Obj ( YOLOv3-tiny-1S)	10.84	89.35
PDF-HMM [49]	39.57	70.68

**Table 8:** Detailed analysis of the phoneme recognition results using the model YOLOv3-tiny-1S.

Speaker/Subset	# Utterance	# Phoneme	# Corr	# Sub	# Del	# Ins	PER
M/C	340	2035	1922	90	23	4	5.7
M/G	340	2035	1887	124	24	2	7.4
Y/C	340	2035	1949	60	26	3	4.4
Y/G	340	2033	1919	85	29	1	5.7
Average							5.8
S.D.							1.2

## Data Availability

Not applicable.
